# Rescue of Early *bace-1* and Global DNA Demethylation by S-Adenosylmethionine Reduces Amyloid Pathology and Improves Cognition in an Alzheimer’s Model

**DOI:** 10.1038/srep34051

**Published:** 2016-09-29

**Authors:** Sonia Do Carmo, Cecilia E. Hanzel, Marie L. Jacobs, Ziv Machnes, M. Florencia Iulita, Jingyun Yang, Lei Yu, Adriana Ducatenzeiler, Marc Danik, Lionel S. Breuillaud, David A. Bennett, Moshe Szyf, A. Claudio Cuello

**Affiliations:** 1 Department of Pharmacology and Therapeutics, McGill University, Montreal, QC, Canada; 2 Rush Alzheimer’s Disease Center, Rush University Medical Center, Chicago, IL, USA; 3 Department of Neurological Sciences, Rush University Medical Center, Chicago, IL, USA; 4 Department of Anatomy and Cell Biology, McGill University, Montreal, QC, Canada; 5 Department of Neurology and Neurosurgery, McGill University, Montreal, QC, Canada

## Abstract

General DNA hypomethylation is associated with Alzheimer’s disease (AD), but it is unclear when DNA hypomethylation starts or plays a role in AD pathology or whether DNA re-methylation would rescue early amyloid-related cognitive impairments. In an APP transgenic mouse model of AD-like amyloid pathology we found that early intraneuronal amyloid beta build-up is sufficient to unleash a global and *beta-site amyloid precursor protein cleaving enzyme 1 (bace-1)* DNA demethylation in AD-vulnerable brain regions. S-adenosylmethionine administration at these early stages abolished this hypomethylation, diminished the amyloid pathology and restored cognitive capabilities. To assess a possible human significance of findings, we examined the methylation at 12 CpGs sites in the *bace-1* promoter, using genome-wide DNA methylation data from 740 postmortem human brains. Thus, we found significant associations of *bace-1* promoter methylation with β-amyloid load among persons with AD dementia, and PHFtau tangle density. Our results support a plausible causal role for the earliest amyloid beta accumulation to provoke DNA hypomethylation, influencing AD pathological outcomes.

Alzheimer’s disease (AD) is a progressive, irreversible neurodegenerative disorder leading to loss of memory and reasoning. Its etiology is complex, resulting from a combination of factors including genetic predisposition, aging, accumulation of amyloid beta (Aβ) peptides, tau hyperphosphorylation, inflammation and oxidative stress^1^,^2^,^3^.

Recently, it has been suggested that epigenetic mechanisms, specifically changes in DNA methylation, which are critical for learning and long-term memory maintenance, contribute to the pathogenesis of several neurodegenerative diseases^4^,^5^,^6^. Global DNA-methylation is reduced in the brain of AD patients^7^,^8^,^9^ and animal models^10^. As a result, there has been growing interest in examining the methylation-status of AD-related genes. Differential methylation has been discovered in humans, in several genes including loci that have been previously associated with AD such as *amyloid precursor protein (app*) and *presenilin 1 (ps1*)^11^,^12^,^13^,^14^,^15^. Studies have also linked hypomethylation of *app* and *ps1* with the aberrant production of Aβ^14^,^16^. However, epigenetic regulation of *beta-site amyloid precursor protein cleaving enzyme 1 (bace-1)* has never been thoroughly studied in the context of the AD pathology, despite its crucial role in Aβ generation.

Although the cause of global hypomethylation in AD remains unclear, low levels of S-adenosylmethionine (SAM) have been found in the brain and cerebrospinal fluid of AD patients^17^,^18^. SAM is a universal and ubiquitous methyl donor that participates in myriad metabolic pathways in the periphery and central nervous system (CNS). SAM depletion *in vitro* and in AD animal models perturbs DNA methylation and increases APP, PS1 and BACE-1 protein expression, stimulating toxic Aβ production and neuropathology^16^,^19^,^20^,^21^.

The growing body of evidence linking epigenetic deregulation and neuropathology has led to the development of therapeutic treatments aimed at increasing DNA methylation or decreasing demethylation. SAM supplementation represents a promising preventative and/or therapeutic option as the compound can be administered orally and crosses the blood-brain barrier^22^. In animal models, SAM supplementation has been effective in treating several neurological conditions^23^,^24^,^25^. SAM treatment has also alleviated AD-like features in models carrying mutations in several AD-relevant genes or that combined mutations in AD genes and dietary deficiencies^26^,^27^,^28^,^29^,^30^.

We combined human data with experiments in a transgenic mouse AD model to test the plausibility of a causal, disease-aggravating, role for DNA methylation in the molecular pathology of AD. We focused on *bace-1* since this gene is critical for Aβ production and triggering of the cascade of events leading to AD pathology. We first investigated, in a transgenic mouse model of AD-like pathology, whether DNA hypomethylation at the *bace-1* gene is a downstream effect of the APP mutation and if so, whether the administration of SAM could reduce this hypomethylation, dampen the amyloid pathology, and offset associated cognitive deficits. We then examined brain DNA methylation at the *bace-1* gene in 740 autopsied subjects from two clinical pathological studies of aging and dementia and measured correlations between methylation levels at several CG sites in the *bace-1* gene with two neuropathological features of AD, β-amyloid load and neurofibrillary tangle density. Our data suggest that *bace-1* hypomethylation is a downstream effector to the initial (preclinical) pathological accumulation of β-amyloid peptides and possibly a mediator of further and accelerated AD pathology. These results point to the possibility that DNA methylation modulators such as SAM might serve as effective treatments for early AD and call for further investigations, developing and testing such approaches.

## Results

### Early burden of Aβ and APP fragments unleashes global brain DNA hypomethylation

**Figure 1:**
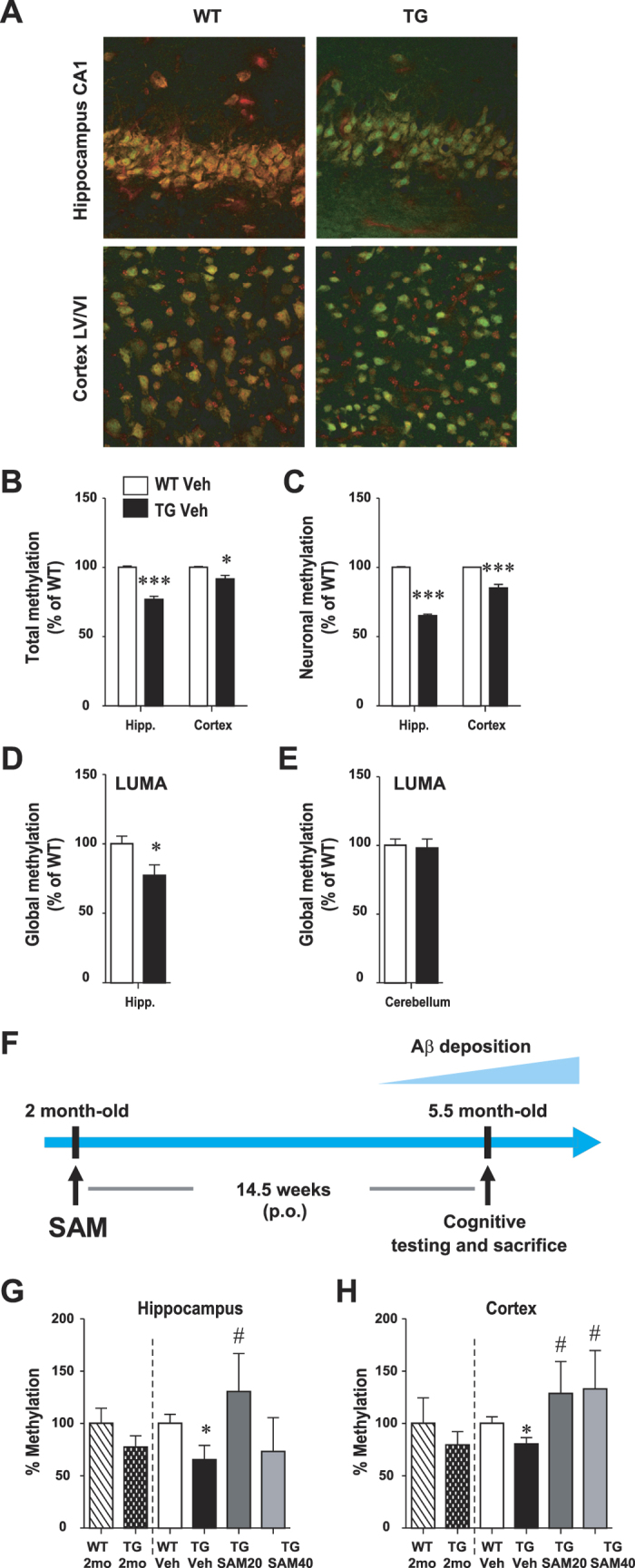
Global hippocampal and cortical DNA hypomethylation and restoration of methylation levels with SAM administration in McGill-Thy1-APP Tg mice. (**A**–**C**) 5-methylcytosine (5-mC) immunofluorescence. Representative images of the hippocampus (CA1 layer) and cerebral cortex (**A**) in 5 month-old animals showing 5-mC immunoreactivity (red labeling) and neuronal nuclei (NeuN, green labeling). Scale bar: 50 μm. (**B**,**C**) Quantification of the total (**B**) and neuronal (**C**) 5-mC relative fluorescence (red) in hippocampus and cortex (WT, n = 3; Tg, n = 3). Methylation is significantly decreased in Tg animals, particularly in the hippocampal neurons. (**D**,**E**) Global methylation levels in the hippocampus (**D**) and cerebellum (**E**) was measured using LUMA. Results are shown as methylation % relative to mean of WT, mean ± SEM. Student’s t-test, *P < 0.05, ***P < 0.001. (**F**) Experimental design of chronic SAM administration. (**G**,**H**) Global methylation was measured by ELISA in the hippocampus (**G**) and cortex (**H**) at 2 months of age, time-point where SAM administration started, and at the end of the treatment. All values are presented as relative percentage compared to WT at 2 months of age (WT 2 mo). Data are expressed as mean ± SEM and analyzed with One-Way ANOVA, followed by Bonferroni post-hoc test; *p < 0.05 vs WT Veh; ^#^p < 0.05 vs Tg Veh.

We first investigated whether DNA methylation alterations are triggered by a genetic mutation that causes the accumulation of APP-derived fragments, leading to AD pathology. We measured global methylation in the McGill-Thy1-APP transgenic (Tg) mouse, which carries a single transgene, the human amyloid precursor protein (APP) containing the Swedish and Indiana mutations under the control of the murine Thy 1.2 promoter^31^. Using 5-Methylcytosine (5-mC) immunohistochemistry (Fig. 1A,B), Luminometric Methylation Assay (LUMA) (Fig. 1D,E) and 5-mC ELISA (Fig. 1G,H), we show that, at 5 months of age, the amyloid pathology was associated with a general decrease in DNA methylation in the hippocampus and cortex. At this time-point, the amyloid pathology is characterized by an accumulation of APP and its derived fragments such as β-CTF, Aβ40 and Aβ42 mainly in the intracellular compartment (Supplementary Fig. S1).

Regional variations in global DNA methylation corresponded well with regional differences in pathology severity, as demethylation within the hippocampus was more pronounced than that observed in the cortex. In this Tg model, intraneuronal Aβ immunoreactivity is observed at one week postnatal, while the first extracellular amyloid plaques develop at 4–5 months of age, starting in the subiculum and spreading to the hippocampus and cortex^31^. Accordingly, we observed strong intraneuronal Aβ immunoreactivity, as well as few diffuse Aβ-immunoreactive amyloid plaques in the hippocampus and cortex at 5.5 months of age (Supplementary Fig. S1). Furthermore, no changes in global methylation were found in the cerebellum (Fig. 1E), a brain region, in this model, devoided of Aβ accumulation^31^ and largely unaffected in AD. In addition, ELISA results suggested that this global hypomethylation may be an early pathological event, as a trend towards reduced methylation was already observed in the hippocampus and cortex of Tg animals at 2 months of age (Fig. 1G,H).

### Aβ-burdened neurons are the prime site for Alzheimer’s-like hypomethylation

Since Aβ first accumulates in the intraneuronal space, and that pyramidal neurons are primary targets in the cortex and hippocampus of AD brains, we investigated the status of DNA methylation in neurons. A hypomethylation, detected by 5-mC immunohistochemistry, was observed in the neuronal population (neuronal nuclear enolase (NeuN)-positive cells) of the hippocampal CA1 region and cortical layers V and VI of the frontal cortex. (Fig. 1A,C) in Tg animals. This hypomethylation was by large more pronounced in neurons (Fig. 1C) than in the overall cell population (Fig. 1B) suggesting that hypomethylation occurs primarily in Aβ-burdened cells.

### Early amyloid peptide accumulation results in significant *bace-1* hypomethylation

**Figure 2:**
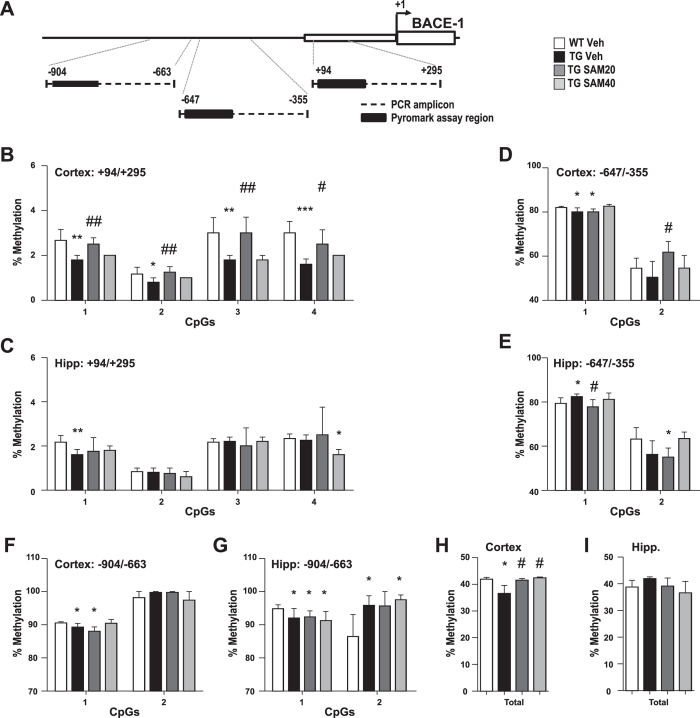
Effect of SAM administration on *bace-1* promoter methylation. (**A**) Methylation was examined in 3 regions of the *bace-1* promoter. Regions amplified by PCR and analysed by pyrosequencing are indicated. Methylation was analysed in region +94/+295 (**B**,**C**), −647/−355 (**D**,**E**), −904/−663 (**F**,**G**) of *bace-1* promoter in the cortex (**B**,**D**,**F**) and hippocampus (**C**,**E**,**G**). Values are given as mean percentage of methylation at individual CpG (**B–G**). (**H**,**I**) Cumulative values of all *bace-1* promoter regions tested were also calculated. Data are presented as mean ± SEM.*p < 0.05, **p < 0.01, ***p < 0.001 vs WT Veh; ^#^p < 0.05, ^##^p < 0.01 vs Tg Veh.

Our genetically triggered AD model exhibits global DNA hypomethylation and increased BACE-1 mRNA, protein levels, and enzymatic activity. We therefore examined by pyrosequencing whether *bace-1* promoter-methylation was altered in our Tg model. Methylation levels were analysed across 3 promoter regions enriched in CpG islands (Fig. 2A). Overall methylation levels in region +94/+295 were generally low in the cortex and hippocampus at about 2% (Fig. 2B,C), as expected for functional promoter sequences. In addition, methylation levels of each individual CpG in region +94/+295 were lower in the cortex of Tg animals (vehicle-treated) compared to their wild-type (WT) counterparts (Fig. 2B). Surprisingly, this difference in methylation was not as evident in the hippocampus of Tg animals and only 1 CpG in this region showed demethylation (Fig. 2C). Regions −647/−355 and −904/−663 displayed higher methylation levels in both cortex and hippocampus, reaching 50–80% to 90–100%, respectively (Fig. 2D–G). In both regions, although present, differences in methylation between WT and vehicle-treated Tg animals were not as marked as in region +94/+295. Finally, when methylation values for all CpGs analysed (region −904 to +295) were combined, global hypomethylation at the *bace-1* promoter became evident in Tg animals compared to their WT littermates in the cortex (Fig. 2H), but not in the hippocampus (Fig. 2I), an aspect likely to be related to the regional progression of the AD pathology and suggesting that most significant impact on DNA hypomethylation might occur at the earlier stages of intracellular Aβ pathology.

### A causal role of hypomethylation in aggravating the AD pathology: Reversal by SAM

We then hypothesized that if hypomethylation is a significant mediator of the AD pathology triggered by Aβ accumulation, then its reversal by the administration of the methyl donor SAM could decrease AD pathology.

We first determined whether chronic administration of SAM could modulate global DNA hypomethylation in AD Tg mice. Tg mice and their WT littermates were treated orally with either 20 or 40 mg/kg of SAM (SAM20 and SAM40) for 3.5 months, starting at 2 months of age (pre-plaque stage) (Fig. 1F). The level of 5-mC, as measured by ELISA, was significantly increased by SAM20 treatment in the hippocampus and cortex of Tg mice (Fig. 1G,H), reaching levels comparable to those observed in WT animals. However, administration of higher doses of SAM (SAM40) failed to restore DNA methylation levels in the hippocampus. Administration of SAM did not have any significant effects on methylation levels in WT animals.

SAM treatment also impacted methylation at the *bace-1* gene. Treatment of Tg animals with SAM20 resulted in increased methylation at each CpG of region +94/+295 in the cortex, reaching levels comparable to those in the WT group (Fig. 2B), whereas treatment with SAM40 resulted only in a trend towards increased methylation. In regions −647/−355 and −904/−663, where differences in methylation between WT and vehicle-treated Tg animals were not as marked as in region +94/+295, treatment with SAM had little-to-no impact on methylation levels. Still, because of a trend towards increased methylation after SAM40 treatment in these regions, when methylation values for all CpGs analysed were combined, it was observed that both doses of SAM reversed cortical hypomethylation, restoring methylation levels to WT values (Fig. 2H). Treatment with SAM had no impact on methylation levels in WT mice (Supplementary Fig. S2). Since oral SAM administration was able to impact both global and *bace-1* promoter DNA methylation, we verified whether SAM was indeed reaching the brain or if the changes were due to peripheral metabolic changes. We detected significant upregulation of SAM cortical levels in Tg animals after treatment with SAM20. This was accompanied by a trend towards increased SAH levels, resulting in an unchanged SAM/SAH ratio (Supplementary Fig. S3).

**Figure 3:**
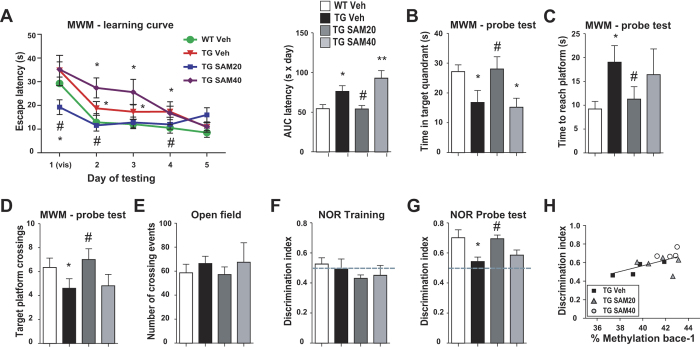
SAM chronic administration reversed the cognitive deficits of McGill-Thy1-APP mice. **(A**–**D**) Deficits in the Morris water maze. (**A**) Latency to locate the hidden platform during the learning phase of the test. Animals were given 4 trials per day for 5 days, with the first 2 trials of day 1 involving a visible platform. Mean latency is calculated as area under the curve (AUC, right panel). (**B–D**) Memory recall was assessed 24 h after completion of the learning phase and time spent in the target quadrant (**B**), time to first reach the platform (**C**) and number of crosses over the annulus that contained the platform (**D**) were analysed. (**E**) Mice were tested for spontaneous activity in open-field. (**F**,**G**) Deficits in Novel Object Recognition test. (**F**) No preference for either of the 2 objects was recorded during the training phase of the task, when 2 identical objects were presented. (**G**) During the probe test, carried 24 h after training, WT mice showed a clear preference for the new object, while the vehicle-treated Tg mice failed to discriminate between the familiar and novel object. Treatment with SAM20 reduced this deficit. The 0.5 discrimination ratio, equivalent to chance is indicated by a dashed line. Data are expressed as mean ± SEM and analyzed with One-Way ANOVA, followed by Bonferroni post hoc test; *p < 0.05 vs WT Veh; ^#^p < 0.05 vs Tg Veh.

We then examined the effects of chronic administration of SAM on cognitive outcomes. In the Morris water maze (Fig. 3A–D), all mice learned to find a hidden platform by spatial navigation with decreasing latency. While vehicle-treated Tg mice had impairments in learning the task compared to their WT littermates, SAM20-treated Tg mice performed significantly better than vehicle-treated Tg animals at days 1, 2 and 4 (Fig. 3A). This difference is further supported by calculating the area under the learning curve (Fig. 3A, right panel). Administration of SAM40, however, did not ameliorate the learning process in Tg mice. Memory retention, as examined by a probe test 24 h after the last day of training, demonstrated that vehicle-treated Tg mice had an impairment in locating the platform quadrant compared to WT. Treatment with SAM20, but not with SAM40, rescued the spatial memory impairment of Tg mice (Fig. 3B–D). Rescue of behavioral deficits in response to SAM20 was also observed in the novel object recognition (NOR) task, which tests episodic memory (Fig. 3F,G). SAM20-treated mice were as efficient as WT mice at discriminating the new object; however, SAM40-treated mice as well as vehicle-treated Tg mice were unable to discriminate the new object (Fig. 3G). Interestingly, in Tg mice, total *bace-1* promoter methylation levels in the cortex (Fig. 2H) positively correlated with behavior outcomes in the NOR task (Fig. 3G,H) (Spearman ρ, 0.5879; P = 0.0173). Treatment with SAM affected neither spontaneous activity, as measured by an open-field behavioral test (Fig. 3E), nor the behavior of WT mice (Supplementary Fig. S2).

**Figure 4:**
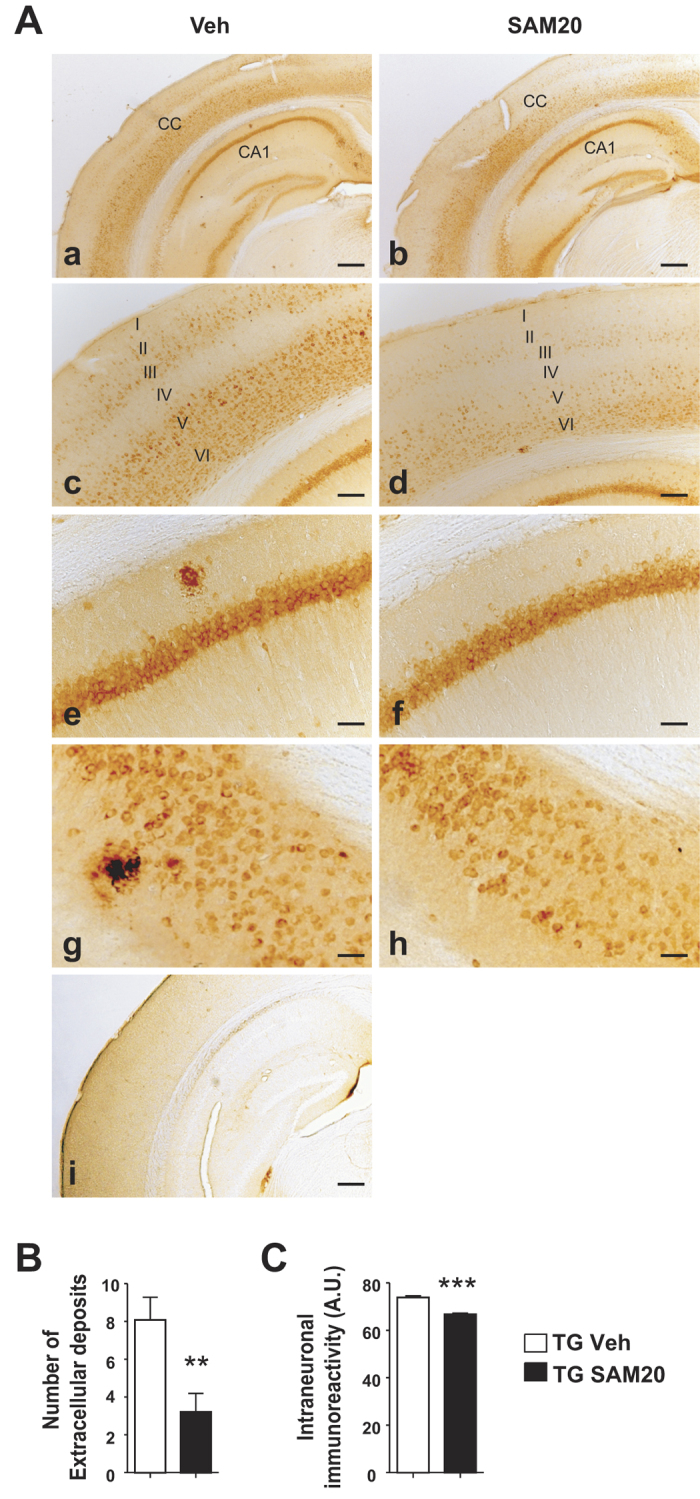
Effect of SAM administration on AD-like amyloid pathology. (**A**) Intense intraneuronal Aβ immunoreactivity is observed in Tg mice throughout the neocortex (a–d), hippocampus (a–f) and subiculum (g,h), as revealed with the anti-Aβ McSA1 monoclonal antibody. Note the absence of McSA1-immunoreactivity in non-transgenic animals (i). Scale bar: a-b, i = 500 μm; c-d = 100 μm; e-h = 50 μm. (**B**) Quantification of the total number of extracellular Aβ-amyloid plaques. (**C**) Quantification of the intensity of intraneuronal McSA1 immunoreactivity in cortical lamina V, presented as arbitrary units (A.U.). Data are expressed as mean ± SEM and analyzed with Student’s t-test. *p < 0.05, **p < 0.01, ***p < 0.001.

**Figure 5:**
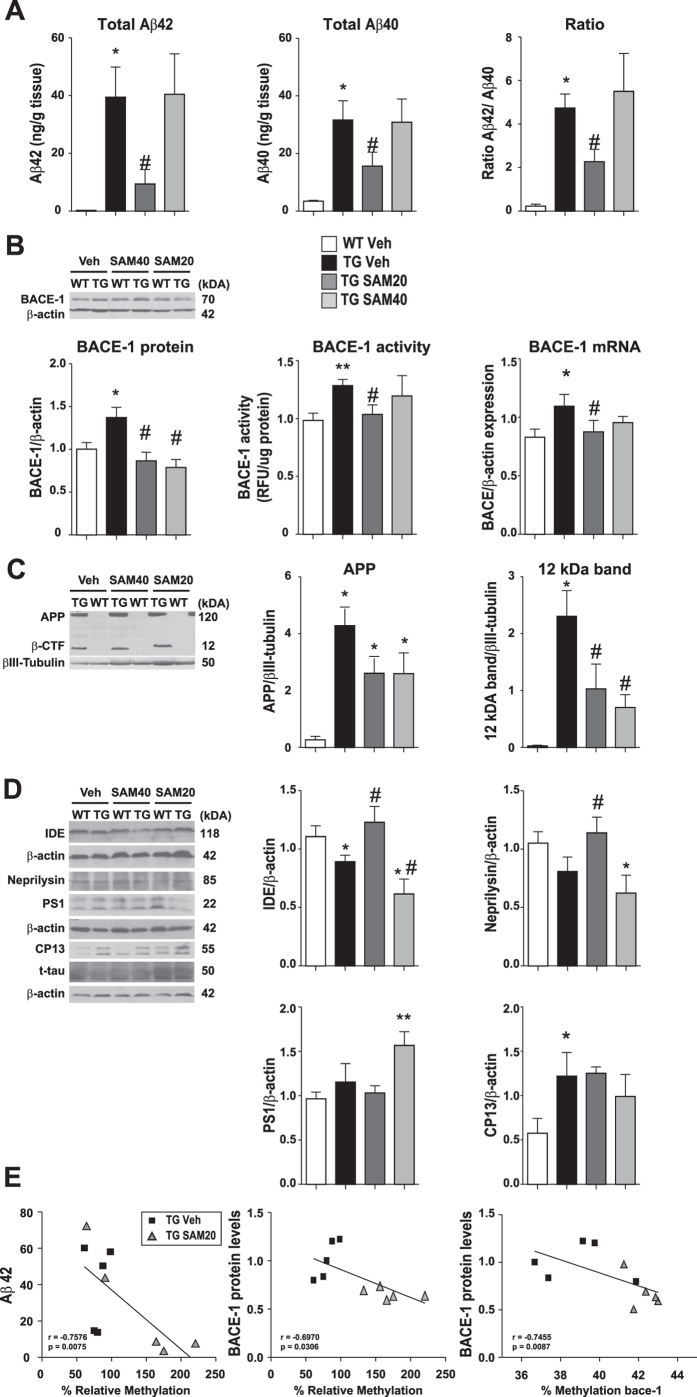
SAM chronic administration impacts the APP processing cascade. Biochemical analyses were performed on cortical homogenates. (**A**) Abundance of human Aβ42 (left) and Aβ40 (middle) peptides was determined by ELISA from guanidine hydrochloride-homogenized tissue. The ratio Aβ42/Aβ40 was also calculated (right). Signals detected in WT animals are considered as assay background. (**B**) The levels of BACE-1 protein, activity and mRNA were determined by western blot, enzymatic assay and qRT-PCR respectively. (**C**) The expression of human APP and the major C-terminal cleavage product of APP (β-CTF) were assessed by western blot (left) using 6E10 antibody. (**D**) The levels of PS1 and of the major Aβ-degrading enzymes IDE and neprilysin were analyzed by western blot. The levels of tau phosphorylated at Ser-202 (CP13) were also determined. Representative blot images of at least three independent experiments are shown. β-actin or βIII-tubulin were used as internal control for quantification and all values are presented as relative levels compared to WT Vehicle. Data are expressed as mean ± SEM and analyzed with One-Way ANOVA, followed by Bonferroni post hoc test; *p < 0.05 vs WT Veh; ^#^p < 0.05 vs Tg Veh. (**E**) Correlations between the cortical levels of Aβ42 (left) and BACE-1 (center) and the levels of global DNA methylation as detected by ELISA. Correlation between BACE-1 protein levels and *bace-1* promoter methylation (right). Only Tg Vehicle and Tg SAM20 were included in the analysis. Spearman’s rank correlation coefficient (ρ) and level of significance (p) are indicated within each graph. Each point represents data from a single animal.

Next, we examined whether improvement in behavior of SAM20-treated mice coincided with a modulation of the amyloid pathology. When compared to vehicle-treated Tg animals, SAM20-treated Tg mice displayed significantly fewer Aβ-plaques and reduced intracellular McSA1-immunoreactive Aβ material in cortex, (Fig. 4), decreased the amounts of human Aβ-peptides (Fig. 5A) and a reduced ratio of Aβ42/Aβ40 peptides, suggesting a reduction of the most toxic Aβ species (Fig. 5A, right panel).

We also determined the status of key proteins involved in the amyloidogenic pathway. Thus the increased BACE-1 mRNA, protein levels and enzymatic activity observed in the Tg animals were all reduced by treatment with SAM20, reaching levels comparable to those observed in WT animals (Fig. 5B). The SAM treatment led to significantly lower levels of the 12 kDa 6E10-immunoreactive band, a likely mix of Aβ trimers and β-CTF fragments (Fig. 5C). SAM20-treated Tg mice also displayed higher levels of insulin degrading enzyme (IDE) and neprilysin, enzymes known to be involved in the clearance of Aβ (Fig. 5D). In addition, SAM20 had no effect on PS1 expression, which is generally not affected in our model (Fig. 5D). Most interestingly, the cortical levels of Aβ42 (Spearman ρ, −0.7576; p = 0.0075) and BACE-1 (Spearman ρ, −0.6970; p = 0.0306) inversely correlated with the levels of DNA methylation as detected by ELISA in vehicle- and SAM20-treated Tg mice. BACE-1 protein levels were also inversely correlated with *bace-1* promoter methylation (Spearman ρ, −0.7475; p = 0.0087) (Fig. 5E).

In contrast, although SAM40-treated Tg mice also displayed significantly lower levels of BACE-1, APP and β-CTF compared to vehicle-treated Tg mice (Fig. 5B,C), their levels of IDE, neprilysin and PS1 (Fig. 5D), of BACE-1 activity (Fig. 5B) and of Aβ peptides (Fig. 5A) were comparable to those of vehicle-treated Tg animals.

Considering that amyloid pathology is accompanied by tau hyperphosphorylation, we examined whether SAM20 was sufficient to impact this pathological marker. However, neither dose of SAM affected tau phosphorylation at Ser202 (Fig. 5D) in this transgenic model, which lacks of overt tau pathology.

### *bace-1* promoter methylation is associated with β-amyloid and PHFtau tangle load and with cognitive decline in human subjects

We examined the methylation level of twelve CpG sites *a priori* located in the promoter region of the *bace-1* gene in cortical samples from 740 donors, of which 235 had no cognitive impairment (NCI), 175 had mild cognitive impairment (MCI) and 311 had AD. Subjects were from two ongoing clinical pathologic studies of aging and dementia: the Religious Orders Study (ROS)^32^ and the Rush Memory and Aging Project (MAP)^33^ (Table S1). In this cohort, all the 12 CpG sites interrogated were extremely hypomethylated as expected for active promoters (Table S2); the methylation β values averaged across the 12 CpGs had a mean of 0.054 (SD: 0.006, range: 0.034–0.079). The average methylation in the *bace-1* promoter was not related to age or education, nor did it differ by sex or clinical diagnosis (all p’s > 0.05). In the regression model adjusted for age, sex and technical confounders, we did not find a significant association of average methylation in the *bace-1* promoter with β-amyloid load (estimate = −0.054, standard error (SE) = 0.043, p = 0.215) and interrogation of individual CpG sites showed that methylation at cg21048949 was nominally associated with lower β-amyloid load (Table 1), but the result did not survive Bonferroni correction for multiple testing (α_adj_ = 0.05/12 = 0.004).

**Figure 6:**
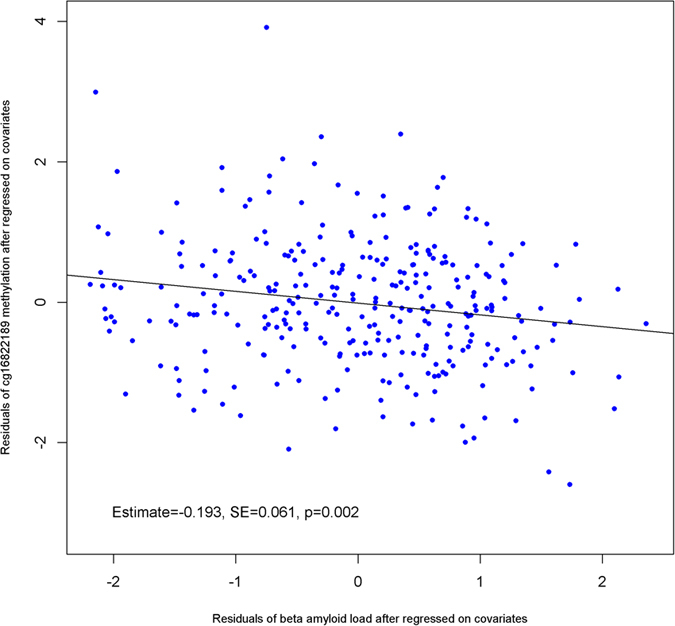
Association of the methylation at cg16822189 located in the *bace-1* promoter with β-amyloid load among persons with AD dementia.

Next, we augmented the regression model by including interaction terms for methylation and diagnosis to determine whether the association of methylation state of the *bace-1* promoter with β-amyloid load differed by the clinical condition. Thus, we found that the F-statistic for the overall interaction was significant (p = 0.033), suggesting the association of average methylation in the human *bace-1* promoter with β-amyloid load differed by clinical diagnosis. In particular, greater methylation was associated with lower β-amyloid load in subjects with AD dementia (estimate = −0.174, SE = 0.062, p = 0.005), while we didn’t find such association in subjects with NCI or MCI (both p’s > 0.05). At the individual CpG level, stratified models showed that, in subjects with AD dementia, greater methylation at cg16822189 was associated with lower β-amyloid load (p = 0.002; Fig. 6). Notably the association remained significant after correcting for multiple testing.

We did not find association of average methylation in the *bace-1* promoter with PHFtau tangles pathology (estimate = 0.001, SE = 0.054, p = 0.990). Further, there was no evidence that the association differed by clinical diagnosis as the interaction was not significant (p = 0.492). At the individual CpG level, cg17007365 showed a positive association with PHFtau tangle density such that greater methylation at the site was associated with higher tangle density (p = 0.001; Table 1). This association remained significant after correcting for multiple testing.

Since methylation at cg16822189 was associated with β-amyloid load and methylation at cg17007365 was associated with PHFtau tangle density, we examined the relationship of these two methylation sites with longitudinal cognitive decline. We did not find an association of methylation at cg16822189 with the rate of decline in global cognition or in any of the five cognitive domains. However, consistent with the findings for PHFtau tangle pathology, methylation at cg17007365 was nominally associated with faster decline in global cognition, as well as episodic memory, semantic memory, and processing speed (Table 2).

## Discussion

Global and gene specific DNA demethylation in Alzheimer’s disease have only been examined in post-mortem brains, making it difficult to assess the contribution of Aβ accumulation to epigenetic changes at early stages. However, the critical question in developing novel therapeutic approaches to AD that target hypomethylation is whether reduced DNA methylation plays a causal role in AD pathology. Global hypomethylation could either represent an alternative route to AD pathology leading to stochastic changes in DNA methylation and expression of genes that could trigger sporadic AD or could serve as an essential downstream component of AD molecular pathology independently of whether it is genetically or epigenetically triggered.

To directly address this question it is essential to use an animal model with a single distinct genetic trigger of AD. Using the McGill-Thy1-APP Tg mouse model^31^, expressing a single transgene, we tested whether mutations in the *app* gene causing an initial neuronal Aβ accumulation would trigger global brain hypomethylation as observed in the post-mortem AD-brain and whether changes in DNA methylation play a causal role in the pathological cascade triggered by Aβ accumulation. Aβ intracellular accumulation is considered an early feature of AD pathology^3^,^34^,^35^,^36^.

We first demonstrated that global hypomethylation is triggered by an early Aβ build-up in both the hippocampus and cortex and that this process is initiated early between two months, when the Aβ pathology is limited to the intracellular compartment, and five months of age, when scattered diffuse amyloid plaques are already present. We also demonstrate that this early hypomethylation, although detected in all brain cells, is more pronounced in neurons. Further to this, our results suggest a threshold of amyloid buildup required to influence 5-mC levels as at two months of age, the difference in methylation between Tg and control animals is not yet significant. These studies strongly suggest that the earliest intraneuronal accumulation of Aβ peptides is sufficient to unleash a generalized DNA demethylation, however, further studies in diverse animal models would be required to define the threshold amount of amyloid required to unleash global hypomethylation,

The presence of an early DNA hypomethylation provides novel clues on the initiation and progression of AD pathology. Changes in DNA methylation in brain have only been reported at stages where extensive amyloid pathology, including thioflavin S-positive mature plaques, was already present^10^,^11^,^30^,^37^,^38^. Furthermore, although neuronal DNA hypomethylation has previously been reported in post-mortem AD^9^, the finding of an early neuronal demethylation in our model reinforces a causal link between Aβ accumulation and deficient DNA methylation, as the amyloid pathology is almost exclusively intraneuronal at this time-point.

Second, we show a demethylation of *bace-1* in the transgenic AD model supporting a role for *bace-1* demethylation in the progression of AD pathology. We consider these results of significance given the critical role of BACE-1 in the production of β-amyloid peptides and hence in AD amyloid pathology. These changes in *bace-1* DNA methylation are associated with expression changes in mRNA and protein expression as well as enzymatic activity of BACE-1. The demethylation in *bace-1* promoter regions triggered by the AD mutations is significant but small in the range of few percent. DNA methylation is a binary signal, a site can either be methylated or not. Thus, 1% methylation indicates that the site is 100% methylated in 1% of cells. This suggests that the changes in methylation occur in a subset of cells in the hippocampus and cortex, most likely in neurons, as BACE-1 is enriched in neurons of the CNS and since neurons play a critical role in Aβ−processing by BACE-1. Our data provide *in vivo* evidence that demethylation is triggered by early Aβ accumulation. We have used a well-accepted method for primer design that assumes that all cytosines except those found at the CpG dinucleotide sequences are unmethylated and are therefore converted to thymidine (T) Fuso and colleagues^39^ have recently shown that these primers might under estimate methylation levels and underestimate methylation differences when the tested region is highly methylated. Since the proximal region of the *bace-1* promoter is extremely hypomethylated this predicted bias should not have affected our analysis. However, our results might have underestimated the differences in DNA methylation between transgenes and controls in the far upstream regions of −647/−355 and −904/−663.

The fact that AD mutation triggers global and site specific DNA demethylation in a critical gene in its downstream molecular pathological pathway is consistent with the hypothesis that DNA demethylation is mediating effects of the APP mutation on AD. In order to test the possible causation, we thought that it was essential to reverse the hypomethylation and measure its impact on the pathological phenotype; for which we resorted to use SAM as the universal methyl donor. SAM is known to be the methyl donor for all DNA methyltransferases catalyzing methylation of DNA and it was also shown to inhibit demethylation and the demethylase enzyme^21^,^40^,^41^, reversing global hypomethylation and modifying gene expression in the CNS and other tissues^42^,^43^. Importantly, SAM has been proven to be non-toxic and to have excellent bioavailability in humans^44^,^45^.

Our results demonstrate that the effects of SAM on the AD-like amyloid pathology are dose-dependent, resulting in better outcomes at the lowest dose tested. Administration of low doses of SAM induced the reversal of DNA hypomethylation (or prevented its progression), including at the *bace-1* promoter. It should be noted however that in contrast to the hippocampus where high dose of SAM has no effect on reversal of DNA methylation, it effects DNA methylation in the cortex. The reason for which global methylation is impacted by high doses of SAM in cortex but not hippocampus remains to be determined in future studies. These results differ from a previous *in vitro* study using human neuroblastoma SK-N-BE cells where treatment with SAM modified BACE-1 mRNA and protein levels in folate and/or vitamin B6 deprivation conditions but did not change *bace-1* methylation status^46^. These differential observations can be ascribed to the well-established fact that cell lines do not necessarily on all circumstances reproduce the physio-pathology of mature CNS in *in vivo* conditions.

This rescue of DNA methylation levels led to decreased accumulation of Aβ material, both in the intracellular compartment and as extracellular plaques, resulting in notable cognitive improvements. In contrast, although higher doses of SAM also impacted cortical DNA methylation, this effect did not translate into decreased production of Aβ-peptides and cognitive improvement. Higher doses of SAM could theoretically trigger excessive or unspecific methylation, which might result in inactivation of essential genes and aversive effects. In support to this, our treatment with low doses of SAM had a remarkable impact on Aβ peptides and plaque accumulation compared to other studies^28^,^29^,^30^. Indeed, while we report reductions of 2.7-fold in the number of plaques, of 4-fold in the levels of Aβ42 peptides and of 2.5-fold in the ratio Aβ42/Aβ40, a study by Fuso and colleagues^28^ reported reductions of almost 2-fold in the percentage of Aβ plaque area and plaque number but no difference in the levels of Aβ42 peptides or in the ratio of Aβ40/Aβ42 using 400 μg of SAM daily. Also, a study by Lee and colleagues^29^ using 400 μg of SAM daily demonstrated substantial reduction in the number of amyloid plaques (7-fold) and a 1.5-fold reduction in the ratio of Aβ/APP. Finally, a study by Li and colleagues^30^ using 13.3 mg SAM/kg/day showed no difference in the levels of Aβ immunoreactivity or Aβ peptides. Also in support to the fact that higher doses do not provide a better outcome on the pathology, a dose-response assay performed in a B-deficient context revealed a lack of significant differences between the intermediate and the highest dose of SAM in terms of PS1 and BACE-1 mRNA expression and consequently in the Aβ40/Aβ42 ratio^28^.

Some of these models carry mutations in several AD-related genes^29^,^30^, while others display accelerated pathology caused by B-vitamin deficiency^28^, which can confound the interpretation of outcomes. Further discrepancies may be explained by differences in the SAM administration paradigm used, including SAM concentration, treatment frequency and pathological stage at the time of treatment initiation. Moreover, the mild effect on Aβ concentration observed in previous studies might be due to higher SAM doses, which are similar to or above our highest SAM concentration. Also in support of a dose-dependent effect of SAM, at high doses, we observed decreased levels of IDE and neprilysin and increased PS1 in SAM40-treated Tg animals, contributing to the accumulation of Aβ-peptides and thus counteracting the beneficial effects resulting from lower doses. These results are consistent with the hypothesis that DNA demethylation facilitates Aβ-triggered AD pathology.

The effect of SAM on brain amyloidosis is twofold. First, SAM treatment decreased *bace-1* mRNA and protein levels and enzymatic activity, decreasing the release of APP cleavage products, such as Aβ40, Aβ42 and β-CTF. This was most likely due to SAM’s effect on the proximal *bace-1* promoter (+94/+295) which actively participates in the regulation of *bace-1* expression, being rich in transcription factor binding sites and showing the highest similarity across mouse, rat and human^47^,^48^. Secondly, the administration of low doses of SAM increased the levels of IDE and neprilysin, contributing to amyloid clearance. Although the effects of SAM on reducing amyloid material has previously been reported^28^,^29^, this is to our knowledge, the first report on SAM’s capacity to lower BACE-1 activity as well as increase neprilysin and IDE levels in a genetically triggered model of AD *in vivo*. It is also among the first reports showing SAM’s capacity to lower BACE-1 protein levels. Another study reported SAM mediated reduction in BACE-1 protein levels under accelerated pathology conditions caused by B-vitamin deficiency but not under normal feeding conditions^28^.

The impact of SAM on behavioral outcomes can be attributed either or both to a modulation of genes involved in learning processes or reduction of toxic Aβ amyloid species. Indeed, SAM is a global methyl group donor and its effects are not limited to amyloid-related genes. As such, it is likely to affect the expression or control of CRTC1 and CRE-regulated proteins related to synaptic plasticity, known to be affected by the early intraneuronal Aβ amyloid pathology^49^. Independently of its effects on gene expression, SAM has been reported to modulate neurotransmitters synthesis^50^, thus controlling neuronal activity and ultimately memory processes. SAM may also control oxidative stress levels through the glutathione system^51^.

The positive effects of SAM on diverse behavior outcomes have also been demonstrated in diverse models of neurological disorders^23^,^24^,^52^. The beneficial effect of SAM on cognition has also been suggested in AD and other neurological diseases^45^,^53^,^54^,^55^ but the small number of patients included in such studies has not provided enough power to reach statistical significance. More recently, treatment of individuals diagnosed with AD with a nutraceutical formulation containing SAM (folate, alpha-tocopherol, B12, SAM, N-acetyl cysteine, acetyl-L-carnitine) under open-label conditions for 12 months resulted in the prevention of cognitive decline^56^.This study represents the first evaluation of SAM cognitive effects in an AD preclinical model triggered by a single transgene (FAD mutated APP) in the absence of exacerbated Aβ production caused by dietary deficiencies. Alternatively, the best therapeutic opportunity could be found in the preclinical stage of AD, for which, unfortunately, we are lacking of unequivocal diagnostic tools.

The potential significance of *bace-1* promoter hypomethylation in AD pathology is further emphasized by its association with clinical-pathological outcomes in human post-mortem brains. In this study, which is the first to explore *bace-1* methylation in such a large population of individuals, we describe the methylation patterns of 12 CpGs located in the *bace-1* promoter and we report that greater methylation in *bace-1* was associated with lower β-amyloid load in subjects with AD dementia. As this association was present in persons with AD dementia but not in individuals with NCI or MCI, who present with lesser β-amyloid load, and since *bace-1* methylation levels are similar across NCI, MCI and AD groups, this suggests that in the human brain very large amounts of Aβ peptides in the form of plaques are needed to impact methylation in the dorsolateral prefrontal cortex. Low levels of methylation in the *bace-1* promoter and similar levels in *bace-1* methylation between AD sufferers and controls were also previously reported in blood DNA^57^.

Another interesting finding is the fact that different CpGs are associated with specific pathological outcomes. While the negative association between *bace-1* methylation and amyloid load in persons with AD dementia was driven by cg16822189, the positive association between *bace-1* methylation and both PHFtau tangle density and cognitive decline was driven by cg17007365, independent of the clinical diagnosis. This observation is in line with other reports where different methylation events in the same gene have opposite associations with outcomes, such as the *APOE* gene^58^. Since PHFtau tangles may be a downstream consequence of *bace-1* epigenetics, through the activation GSK3 by Aβ^59^, it can be hypothesized that low methylation at cg17007365 allows the binding of a factor acting as a repressor of tangle formation.

Although our results provide a plausible causal link between Aβ accumulation and DNA hypomethylation *in vivo*, the detailed mechanisms involved in this significant process would require further investigation. It is conceivable that multiple pathways could mediate the effects of Aβ on DNA methylation.

Our results are consistent with the attractive possibility that SAM has a preventative benefit. SAM is commonly used as a nutritional supplement, however as it is sold over the counter, there is no simple record of its use in the population. As well, there are very few clinical trials using SAM as a monotherapy. Most commonly, SAM is used as part of a nutraceutical formulation (as in ref. 56) thus making it difficult to separate the effects of each component. Perhaps, as SAM is prescribed in the treatment of depression^60^ and chronic liver disease^61^, these populations could serve as a starting point for further investigations on beneficial effects on reducing risk of AD.

In summary, we have shown experimentally that global CNS hypomethylation associated with Aβ build-up can be modulated in a timely manner at adequate doses with SAM administration. Chronic therapy, starting at early stages, was sufficient to improve cognitive outcomes. The positive effects of SAM on preventing amyloid accumulation and subsequent memory deficits were mediated through the restoration of *bace-1* promoter methylation. We have also shown that *bace-1* hypomethylation is relevant in the human context as we observed an association between specific CpGs methylation and pathology (amyloid and tangle load) or cognitive function. Most importantly, low doses of SAM resulted in better outcomes than higher doses suggesting a fine regulation of DNA methylation/demethylation homeostasis in the CNS. We postulate that incipient Aβ pathology is sufficient to provoke a global DNA hypomethylation in the CNS activating disease-aggravating genes such as *bace-1,* thus accelerating the AD neuropathology. These data support a causal role for DNA demethylation in AD pathology, independently of the possibility that epigenetic events could unleash AD pathology (to be proven). Given that SAM is safe and well tolerated in humans, our results could have translational value for individuals at high risk of developing AD.

## Methods

### Animals and S-adenosylmethionine Treatment

McGill-Thy1-APP Tg mice express human APP with Swedish and Indiana mutations under control of the murine Thy 1.2 promoter^31^. A first, untreated group of Tg and non-transgenic wild type (WT) mice was sacrificed at 2 and 5 months of age to evaluate DNA methylation levels at pre- and early post-plaque stages respectively (n = 3–5 animals per genotype per time-point). A second group of Tg (n = 21) and WT (n = 24) mice was divided based on genotype and treatment condition. Animals received orally vehicle, SAM low dose (20 mg/kg; SAM20) or high dose (40 mg/kg; SAM40) (Life Science Laboratories, Lakewood, NJ) (Table S3), 3 times per week from the age of 2 months (before the presence of Aβ plaques) until the age of 5.5 months. All procedures were carried out in accordance with the guidelines set down by the Canadian Council of Animal Care and were approved by the Animal Care Committee of McGill University.

### Behavioral Tests

Novel object recognition (NOR) and Morris water maze (MWM) tests were performed as previously described^31^.

### Tissue Processing

Immediately following the MWM probe trial, mice were deeply anesthetized before trans-cardiac perfusion with ice-cold saline solution (pH 7.4). The left hemisphere was dissected and frozen at −80 °C until processing for biochemistry and molecular analyses. For 5-mC and NeuN immunohistochemistry, the right hemisphere was sliced into 16 μm-thick coronal sections with a cryostat (CM3050 S, Leica, Wetzlar, Germany). For McSA1 immunohistochemistry, the right hemisphere was post-fixed in 4% paraformaldehyde (PFA) in 0.1 M phosphate buffer (PB, pH 7.4) and sectioned into 40 μm-thick coronal sections with a freezing sledge microtome (SM 2000R, Leica, Wetzlar, Germany) at −20 °C^31^,^62^,^63^.

### Western Blotting

Cortical samples (~20 mg) were manually homogenized in 8 volumes of 1X CLB buffer (Cell Signaling, Beverly, MA, USA) complemented with protease inhibitors (Roche, Mannheim, Germany), phosphatase inhibitors (Roche, Mannheim, Germany), and 1 mM phenylmethylsulfonyl fluoride (PMSF). Total protein content was assessed using a Bradford-based protein assay (Bio-Rad, Mississauga, ON). Cortical samples (15–50 μg) were subjected to Western blotting using standard protocols and the primary antibodies listed in Table S4. Then, membranes were incubated with the appropriate horseradish peroxidase-conjugated secondary antibodies (1:5000, Jackson ImmunoResearch Laboratories, West Grove, PA, USA). Immunoreactivity was visualized by chemiluminescence (Pierce, Thermo Scientific, Waltham, MA, USA). Signal intensities were quantified from film exposures (X-Omat LS, Kodak, Rochester, NY) using GelPro Analyzer software (Media Cybernetics, Rockville, MD, USA) and normalized to β-Actin or βIII-Tubulin.

To evaluate APP and its derived products, 100 μg of protein was loaded on Tris-Tricine 10−20% polyacrylamide gradient gels (Bio-Rad Laboratories, Mississauga, ON) and transferred to nitrocellulose membranes (BioRad, Mississauga, ON). Membranes were incubated in boiling PBS for 5 min, and blocked for 2 hours in 10% milk in TBS-T (Tris-buffered saline: 20 mM Tris, 150 mM NaCl, pH 7.6, containing 0.1% Tween 20). followed by overnight primary incubation (6E10), secondary incubation, development and analysis.

### Immunohistochemistry

For 5-mC and NeuN Double Labelling, 16 μm coronal sections were thaw-mounted onto gelatin subbed slides, fixed in 4% PFA (10 min) and processed for immunohistochemistry. Images from cerebral cortex (laminas V and VI) and hippocampus (CA1, CA2, CA3 layers) were obtained using a Zeiss LSM 510 confocal microscope (Zeiss, Canada, Toronto, ON, Canada) and analysed using the Image-ProPlus Software (Media Cybernetics, Rockville, MD, USA).

Free floating immunohistochemical staining using McSA1 (MediMabs, Montreal), a mouse monoclonal antibody recognizing the first 12 amino acids of the Aβ sequence^62^ (Table S4), was performed as previously described^63^. McSA1-immunoreactive plaques were counted by a single experimenter blinded to experimental conditions. To measure the total amount of McSA1 immunoreactive signal within cortical lamina V neurons, a region of interest (ROI) was hand-drawn around randomly selected neuron bodies and integrated pixel intensity values were determined for each ROI, using the Image J software (http://rsb.info.nih.gov/ij/).

### Aβ40 & Aβ42 ELISAs

Total human Aβ40 and Aβ42 levels were quantified from cortical homogenates by ELISA (Invitrogen, Carlsbad, CA, USA). Each sample (10–15 mg) was homogenised in 8 x (v/w) cold 5 M guanidine HCl/50 mM Tris HCl and incubated 4 hours at room temperature. The resulting samples were further diluted 1:10 for Aβ40 and 1:50 for Aβ42 in Dilution Buffer and tested in duplicate. The amount of Aβ was extrapolated from a calibration curve of synthetic human Aβ40 and Aβ42. The data were normalized by amount of tissue (ng Aβ/g tissue).

### SAM & SAH ELISAs

SAM and SAH levels were quantified from cortical homogenates by ELISA (myBioSource, San Diego, CA, USA). Each sample (10–15 mg) was homogenised in 5 volumes (v/w) of 1X CLB buffer (Cell Signaling, Beverly, MA, USA) complemented with protease inhibitors (Roche, Mannheim, Germany). Total protein content was assessed using a Bradford-based protein assay (Bio-Rad, Mississauga, ON). The resulting samples were further diluted 1:25 in Sample Diluent and tested in duplicate. The amount of SAM and SAH were extrapolated from calibration curves of synthetic SAM and SAH standards. The data were normalized by amount of tissue (ng/g tissue).

### BACE-1 Activity Assay

BACE-1 activity was measured using the Fluorometric Beta Secretase Activity Assay (Abcam, England, UK) according to manufacturer’s instructions. Cortical tissue (10 mg) was homogenized in 12 x (v/w) of ice-cold Extraction Buffer and cleared by centrifugation. 50 μl of the resultant samples were loaded into a black 96-well microplate and the fluorogenic substrate was added in the dark. The reaction was incubated at 37 °C for one hour in the dark, and the fluorescent signal produced by cleavage of the peptide by β-secretase was measured using a Fluostar Optima microplate reader (BMG Labtech GmbH, Ortenberg, Germany) with 355 nm excitation and 520 nm emission wavelengths. A blank well, a positive control of active beta secretase in Extraction Buffer and a negative control consisting of beta secretase inhibitor added to the positive control were included.

### Genomic DNA extraction

Cortical and hippocampal tissue (200 mg) was homogenized and digested with 400 μg/ml Proteinase K (Roche, Germany) in 250 μl Lysis Buffer (10 mM Tris, pH 8.0, 400 mM NaCl, 2 mM EDTA, 1% SDS) at 56 °C for 16 h. Samples were treated with RNase A (50 U/mg; 30 min; Roche, Indianapolis, IN), cleaned with phenol-chloroform (1:1) and precipitated with ethanol. The DNA pellet was re-dissolved in 50 μl TE buffer (10 mM Tris-HCL, 1 mM EDTA). DNA purity and concentration were assessed using spectrophotometric analysis. DNA integrity was verified on a 1% agarose gel.

### Luminometric Methylation Assay (LUMA)

LUMA assay was conducted on 1 μg genomic DNA as previously described^64^,^65^. Genomic DNA (1 μg) was digested by methylation sensitive (10U HpaII, New England Biolabs (NEB), Ipswich, MA) or insensitive (10U MspI, NEB) restriction enzymes in combination with an internal control restriction enzyme (10U EcoRI, NEB). The extent of cleavage was determined by a bioluminometric polymerase extension assay based on a four-step pyrosequencing reaction (PyroMark q24, Qiagen, Valencia, CA, USA). Normalized methylation levels were calculated using the following formula: Methylation (%) = 1 − [(HpaII/EcoRI)/(MspI/EcoRI)] × 100. All samples were run in triplicate and analysis of each tissue sample was carried out in three independent LUMA analyses.

### Global DNA Methylation Assay

Global DNA methylation in cortex and hippocampus was assayed by ELISA using the Imprint® Methylated DNA Quantification Kit (Sigma-Aldrich, St-Louis, MO). 100 ng of DNA were diluted in 30 μl binding buffer and added to the wells. After incubation with capture and detection antibodies, the methylated DNA was detected colorimetrically by reading the absorbance at 450 nm. Quantification of global DNA methylation in samples was performed using a standard curve of methylated control DNA. All samples were analyzed in duplicate.

### Pyrosequencing

Bisulfite conversion of 1 μg genomic DNA was performed with the EZ DNA Methylation-Gold Kit (Zymo Research, Irvine, CA, USA) according to the manufacturer’s protocol, and stored at −20 °C until use. Bisulfite converted DNA was eluted in 10 μl of elution buffer, diluted 1:3 with water and 2 μl of diluted DNA were used for PCR. Each 50 μl PCR reaction contained 2.5 mM MgCl_2_ and 0.4 μM each of forward and reverse primers designed with PyroMark Assay Design Software 2.0 (Qiagen). Primers used to amplify CpG-rich regions of the mouse *bace-1* promoter are detailed in Table S5. The reverse primer was biotinylated at its 5’ end to create a single-stranded DNA template. The PCR conditions were as follows: 98 °C for 15 s; 40 cycles of 98 °C for 10 s, 54 °C for 30 s, 72 °C for 30 s, and a final extension at 72 °C for 5 min. Then, 25 μl of PCR product were immobilised on streptavidin beads (GE Healthcare Lifesciences, Baie d’Urfe QC) and pyrosequencing was performed using the PyroMark Q96 ID System (Qiagen)^66^.

### RNA Extraction and Quantitative Reverse-Transcription PCR (qRT-PCR)

Total mRNA was extracted from 20 mg hippocampal tissue using the Qiagen RNeasy Mini Kit (Qiagen). RNA was then retrotranscribed to cDNA using an oligo(dT) primer and the Qiagen Omniscript Reverse Transcription Kit (Qiagen), including an additional DNase I digestion step. Quantification of transcript expression was assessed by qRT-PCR with EvaGreen® (MBI EVOlution EvaGreen qPCR Mix, Montreal Biotech Inc.) using the Illumina Eco Instrument and Software (Illumina Inc., San Diego, USA). Expression of each gene was normalized to the housekeeping gene β-actin. For the list of primers used, see Table S5.

### Human subjects

DNA methylation data for the human *bace-1* promoter was obtained from frozen dorsolateral prefrontal cortex of 740 autopsied subjects from two community-based cohort studies of aging and AD, the Religious Orders Study (ROS)^32^ and the Rush Memory and Aging Project (MAP)^33^. Older persons enroll without known dementia and agree to annual clinical evaluation and brain donation at the time of death. Participants sign an informed consent and Anatomical Gift Act. The studies were in accordance with the latest version of the Declaration of Helsinki and were approved by the Institutional Review Board of Rush University Medical Center. Characteristics of the study subjects are shown in Table S1.

### Annual cognitive assessment

All participants underwent comprehensive annual cognitive assessment, as previously described^67^. Seventeen tests were common in ROS and MAP and were used to obtain a composite measure for global cognition and five domain specific measures for episodic memory (7 tests), semantic memory (3 tests), working memory (3 tests), processing speed (2 tests), and visuospatial ability (2 tests). Briefly, raw scores of each test were first converted to z scores using the mean and standard deviation (SD) from the baseline evaluation of all participants in both cohorts, which were then averaged across tests to yield corresponding summary measures. Of the 740 subjects, the length of follow-up had an average of 6.1 years (SD = 3.9) and was up to 16 years.

### Clinical consensus diagnosis of AD dementia

After a participant died, all available cognitive and select clinical data were reviewed by a physician with expertise in dementia, and a summary diagnostic opinion was provided regarding dementia status at the time of death, blinded to the post-mortem data^68^. AD dementia was diagnosed following the guidelines of the joint working group of the National Institute of Neurological and Communicative Disorders and Stroke and Alzheimer’s Disease and Related Disorders Association^69^. Participants with cognitive impairment but did not meet dementia criteria were diagnosed with mild cognitive impairment (MCI). Case conferences involving at least one neurologist and one neuropsychologist were used for consensus on selected cases. Of the subjects included in the analysis, 43.1% (N = 311) had AD dementia, 24.3% (N = 175) had MCI and 32.6% (N = 235) were cognitive normal (NCI).

### Quantification of β-amyloid load and PHFtau tangle density

Uniform examination for AD pathology including β-amyloid and PHFtau tangles was conducted at autopsy, as previously described^70^,^71^. Blocks of 8 pre-selected brain regions (hippocampal, entorhinal, midfrontal, inferior temporal, calcarine, superior frontal, anterior and cingulate gyrus) were cut into 20 μm sections for immunohistochemistry. β-amyloid was labeled with an N-terminus–directed monoclonal antibody (10D5; Elan, Dublin, Ireland; 1:1,000) and was quantified using an automated, multistage computational image analysis. Percent area positive for β-amyloid was obtained for each region, square root transformed and then averaged across regions to derive a measure for β-amyloid load. PHFtau was labeled with an antibody specific for phosphorylated tau (AT8; Innogenetics, San Ramon, CA; 1:1,000). PHFtau positive tangles per mm2 was quantified using the stereological mapping station, which were averaged within and across brain regions to obtain a measure of PHFtau tangle density. Since the measure was right skewed, we applied square root transformation prior to the analysis.

### DNA Methylation

Detailed information on DNA methylation data collection from post-mortem human brain has been published elsewhere^11^. Briefly, 100 mg sections of frozen dorsolateral prefrontal cortex were thawed on ice with the gray matter dissected from the white matter. DNA extraction was performed using the Qiagen (cat: 51306) QIAamp DNA mini protocol. DNA methylation profile data was generated using the Broad Institute’s Genomics Platform for the Illumina InfiniumHumanMethylation450 bead chip assay. The platform captured approximately half a million CpG sites from human reference genome. The DNA methylation level at each CpG site was presented as a beta value (β), calculated as the ratio of signal from the methylated probe to the sum of both methylated and unmethylated probes, and ranged from 0 (0% methylation or completely unmethylated) to 1 (100% methylated). For the purpose of this study, we focused on the 12 CpG sites located in the promoter region of the *bace-1* gene (Table S2).

### Data Analysis

Bivariate associations of DNA methylation with demographics and clinical diagnosis were examined using Spearman correlation, t-test and analysis of variance, as appropriate. To assess the methylation association with β-amyloid load, we conducted a series of multivariable linear regression analyses with β-amyloid load as the continuous outcome and DNA methylation level as the predictor. A significant and negative regression coefficient would suggest that greater methylation was associated with less β-amyloid load. Both mean methylation averaged across the 12 CpGs as well as methylations at individual sites were examined. All the models were adjusted for age, sex and potential technical confounders including bisulfite conversion efficiency and batch. We added interaction terms for DNA methylation and clinical diagnosis to examine whether the association of DNA methylation differed between subjects with NCI, MCI and AD dementia. The presence of interaction was further investigated by using stratified models where we tested the methylation association with β-amyloid for each diagnostic group separately. Next, we examined the methylation associations with PHFtau tangle pathology where we replaced the outcome with PHFtau tangle density. Finally, we examined the influence of DNA methylation at the *bace-1* promoter with cognitive decline many years prior to death using a series of linear mixed models. In these models, the rate of decline in cognition was modeled as a linear function of DNA methylation. Person-specific deviations from the mean change in cognition were estimated by random effect. These models used annual composite cognitive measures as continuous longitudinal outcomes and included terms for methylation level, time in years prior to death, as well as a methylation by time interaction. The coefficient for the interaction estimated the methylation association with rate of change in cognition. The models were adjusted for age, sex, education, bisulfite conversion efficiency and batch. Notably, these models are chosen tailored to the corresponding outcome measure. Compared to nonparametric approaches, linear regression and linear mixed models are more flexible in the adjustment for confounding factors. Multivariable linear regression analyses in particular are routinely employed in our prior work to examine methylation associations with age and AD pathologies in genome-wide DNA methylation scans^11^,^72^ as well as candidate loci queries^15^,^73^,^74^.

All the statistical analyses were performed using SAS software, version 9.3 (SAS Institute, Cary, NC) and the program R (www.R-project.org). Unless otherwise noted, statistical significance was set at a nominal level of α = 0.05.

Results in mouse are expressed as mean ± SEM. Experimental differences were assessed by Student’s t-test, one- or two-way analysis of variance (ANOVA) followed by Bonferroni post-hoc comparisons, and Spearman correlation analysis using GraphPad Prism 5 software (GraphPad Software, San Diego, CA). Significance was set at p < 0.05.

## Additional Information

**How to cite this article**: Do Carmo, S. *et al*. Rescue of Early *bace-1* and Global DNA Demethylation by S-Adenosylmethionine Reduces Amyloid Pathology and Improves Cognition in an Alzheimer’s Model. *Sci. Rep.* **6**, 34051; doi: 10.1038/srep34051 (2016).

## Supplementary Material

Supplementary Information

## Figures and Tables

**Table 1:** Association of the methylation at the human *bace-1* promoter with β-amyloid load and PHFtau tangle density.

Predictor	β-Amyloid Load			PHFtau Tangle Density		
	Parameter Estimate	Std Error	p-value	Parameter Estimate	Std Error	p-value
cg01025770	−0.0113	0.0405	0.7807	−0.0102	0.0506	0.8405
cg02062003	−0.0111	0.0404	0.7829	−0.0367	0.0504	0.4673
cg07119404	0.0451	0.0412	0.2736	0.0369	0.0514	0.4735
cg07619960	0.0132	0.0403	0.7425	−0.0450	0.0502	0.3710
cg14112985	0.0148	0.0422	0.7263	−0.0564	0.0527	0.2847
cg15427448	−0.0215	0.0419	0.6082	−0.0091	0.0523	0.8627
cg16822189	−0.0742	0.0393	0.0591	−0.0714	0.0490	0.1459
cg17007365	0.0131	0.0392	0.7382	0.1582	0.0486	0.0012*
cg21048949	−0.0970	0.0404	0.0167	−0.0779	0.0506	0.1239
cg22261612	0.0220	0.0394	0.5771	0.0424	0.0491	0.3882
cg23435082	−0.0539	0.0400	0.1787	0.0599	0.0499	0.2308
cg26462656	−0.0733	0.0425	0.0848	−0.0452	0.0531	0.3944

The estimate represents the change in pathology (amyloid or tangles) with one unit increase in methylation. Estimates, standard errors, and p-values at each row came from separate multivariable linear regression models with either β-amyloid load or PHFtau tangle density as the continuous outcome and DNA methylation as the predictor, adjusted for age, sex, bisulfite conversion efficiency and batch. Note that although methylation at cg21048949 was nominally associated with lower β-amyloid, the result did not survive Bonferroni correction for multiple testing.

**Table 2:** Association of the methylation at cg17007365 located in the human *bace-1* promoter with decline in cognition.

	Parameter Estimate	Std Error	p-value
Global cognition	−0.0118	0.0050	0.0182*
Episodic memory	−0.0135	0.0055	0.0151*
Semantic memory	−0.0171	0.0064	0.0078*
Working memory	−0.0069	0.0048	0.1533
Processing speed	−0.0139	0.0057	0.0148*
Visuospatial ability	−0.0087	0.0050	0.0788

The estimate represents the change in cognitive function with one unit increase in methylation. Estimates, standard errors, and p-values at each row came from separate linear mixed models with annual cognitive measures as the longitudinal outcomes and DNA methylation as the predictor, adjusted for age, sex, education, bisulfite conversion efficiency and batch.
