# Supplementary material for: Rescue of Early bace-1 and Global DNA Demethylation by S-Adenosylmethionine Reduces Amyloid Pathology and Improves Cognition in an Alzheimer’s Model
Source: Sci Rep. 2016 Sep 29;6:34051. doi: 10.1038/srep34051 (PMC5041108; doi:10.1038/srep34051)
Supplement: Supplementary Information [file srep34051-s1.pdf]

Supplementary Information for

Rescue of Early *bace-1* and Global DNA Demethylation by S-Adenosylmethionine Reduces Amyloid Pathology and Improves Cognition in an Alzheimer's Model

Sonia Do Carmo[1], Cecilia E. Hanzel[1], Marie L. Jacobs[1], Ziv Machnes[1], M. Florencia Iulita[1], Jingyun Yang[4,5], Lei Yu[4,5], Adriana Ducatenzeiler[1], Marc Danik[1], Lionel S. Breuillaud[1], David A. Bennett[4,5], Moshe Szyf[1], A. Claudio Cuello[1,2,3*]

[1] Department of Pharmacology and Therapeutics, McGill University, Montreal, QC, Canada.
[2] Department of Anatomy and Cell Biology, McGill University, Montreal, QC, Canada.
[3] Department of Neurology and Neurosurgery, McGill University, Montreal, QC, Canada.
[4] Rush Alzheimer's Disease Center, Rush University Medical Center, Chicago, IL, USA.
[5] Department of Neurological Sciences, Rush University Medical Center, Chicago, IL, USA.

* Correspondence should be addressed to:

A. Claudio Cuello, Department of Pharmacology and Therapeutics, McGill University, 3655 Promenade Sir-William-Osler, Room 1210, Montreal, Quebec, Canada, H3G 1Y6; e-mail: claudio.cuello@mcgill.ca

Table S1. Demographic information of the cases used in this study. Participants were from two ongoing clinical pathologic studies of aging and dementia: the Religious Orders Study (ROS) and the Rush Memory and Aging Project (MAP).

		**NCI (*n* = 235)**	**MCI (*n* = 175)**	**AD (*n* = 311)**
**Age at death (years)**	Age range	66.2 - 101.2	66.0 - 106.5	70.3 - 108.3
	Mean ± SEM	85.3 ± 0.43	87.9 ± 0.49	90.3 ± 0.34
**Gender (F/M)**		147/88	106/69	207/104
**Cortical area occupied by amyloid (%)**	Mean ± SEM	2.2 ± 0.18	2.9 ± 0.27	4.8 ± 0.22***
**Tangles density (mm^2)**	Mean ± SEM	2.8 ± 0.22	4.5 ± 0.39*	10.44 ± 0.58***
**Final Cognitive Diagnosis (cogdx 1-6)**	Range	1	2-3	4-5

ANOVA Dunnett vs control

Cogdx:

1: NCI, No cognitive impairment (No impaired domains); 2: MCI, Mild cognitive impairment (One impaired domain) and NO other cause of CI; 3: MCI, Mild cognitive impairment (One impaired domain) AND another cause of CI; 4: AD, Alzheimer's disease and NO other cause of CI (NINCDS PROB AD); 5: AD, Alzheimer's disease AND another cause of CI (NINCDS POSS AD); 6: Other dementia, Other primary cause of dementia.

Table S2. Methylation levels of the 12 CpGs at the human *bace-1* promoter included in this study.

Variable	N	Mean	Std Dev	Minimum	Maximum
cg01025770	740	0.0625	0.0107	0.0259	0.1168
cg02062003	740	0.0633	0.0105	0.0310	0.1066
cg07119404	740	0.0656	0.0097	0.0372	0.1103
cg07619960	740	0.0730	0.0145	0.0317	0.1281
cg14112985	740	0.0441	0.0094	0.0089	0.0913
cg15427448	740	0.0085	0.0063	0	0.0365
cg16822189	740	0.0379	0.0147	0	0.0974
cg17007365	740	0.0402	0.0115	0.0026	0.0855
cg21048949	740	0.0471	0.0112	0.0110	0.0926
cg22261612	740	0.0977	0.0180	0.0362	0.1525
cg23435082	740	0.0916	0.0193	0.0447	0.1799
cg26462656	740	0.0136	0.0134	0	0.0717

Table S3: Description of experimental groups treated from age 2 to 5.5 months with SAM or Vehicle.

Genotype	Treatment	Description	Number and Sex of Animals
WT	Vehicle	WT Veh	n = 6; 2M + 4F (biochemistry/methylation)<br>n = 3; 2M + 1F (IHC)
WT	SAM 20 mg/kg	WT SAM20	n = 6; 3M + 3F (biochemistry/methylation)<br>n = 3; 2M + 1F (IHC)
WT	SAM 40 mg/kg	WT SAM40	n = 6; 3M + 3F (biochemistry/methylation)
TG	Vehicle	TG Veh	n = 5; 1M + 4F (biochemistry/methylation)<br>n = 3; 1M + 2F (IHC)
TG	SAM 20 mg/kg	TG SAM20	n = 5; 2M + 3F (biochemistry/methylation)<br>n = 3; 1M + 2F (IHC)
TG	SAM 40 mg/kg	TG SAM40	n = 5; 2M + 3F (biochemistry/methylation)

Table S4: Primary antibodies used for Western blotting and immunohistochemistry.

Antibody	Dilution	Reference
Western blotting		
6E10 (APP and products)	1:1000	Covance, Signet Laboratoires (#39320), Princeton, NJ, USA
BACE1	1:1000	Cell Signalling (#5606), Beverly, MA, USA
BDNF	1:1000	Santa Cruz Biotechnologies (#sc-546), TX, USA
CP13 (Tau)	1:1000	Generous gift from Dr. Davies (Herskoutis & Davies, 2006)
IDE	1:1000	Calbiochem/ EMD Millipore (#PC730), MA, USA
PSN1	1:500	Calbiochem/ EMD Millipore (#AB5757), MA, USA
Neprilysin	1:2000	R&D Systems, Minneapolis, MN, USA
β-actin	1:5000	Abcam (#ab8227), England, UK
βIII-tubulin	1:2000	Promega (#G7121), Madison, WI, USA
t-tau (Tau 5)	1:1000	Calbiochem (#577801), MA, USA
Immunohistochemistry		
McSA1	1:400	Medimabs, Montreal, QC
5-methylcytosine	1:500	Calbiochem (#MABE527), MA, USA
NeuN-Alexa Fluor 488	1:1000	Calbiochem (#MAB377), MA, USA
Alexa Fluor 568	1:600	Invitrogen/Life Tech. (#A-11031) Burlington, ON

Table S5: Primers used for *bace-1* pyrosequencing and qRT-PCR analyses in mouse (5’-3’).

**Primers used for *bace-1* pyrosequencing**		
Region -904/-663:		
Forward	TGG GAA GTA ATT AGA TGG GTT ATG AGG	
Reverse	CTT CTA TAC CTA AAT CCC TTT ATC TTT CT	
Sequencing	AGA TGG GTT ATG AGG A	
Region -647/-355:		
Forward	AGT TGG GGT AGG TTA AAT GT	
Reverse	AAA CAC CTA CCT TTA ATC TCA AC	
Sequencing	ATG TTT TGA ATA GAA GAG G	
Region +94/+295:		
Forward	AGT TGG GAG TTG GAT TAT GGT	
Reverse	CCA AAA AAC CCA ACT ACA TCT AA	
Sequencing	GAT TAT GGT GGT TTG AG	
**Primers used for qRT-PCR analyses**		
Gene:	Forward Sequence:	Reverse Sequence:
β-Actin	CTA AGG CCA ACC GTG AAA AG	ACC AGA GGC ATA CAG GGA CA
bace-1	TGC AAG GAG TAC AAC TAC GAC	CAT CCG GGA ACT TCT CCG TC

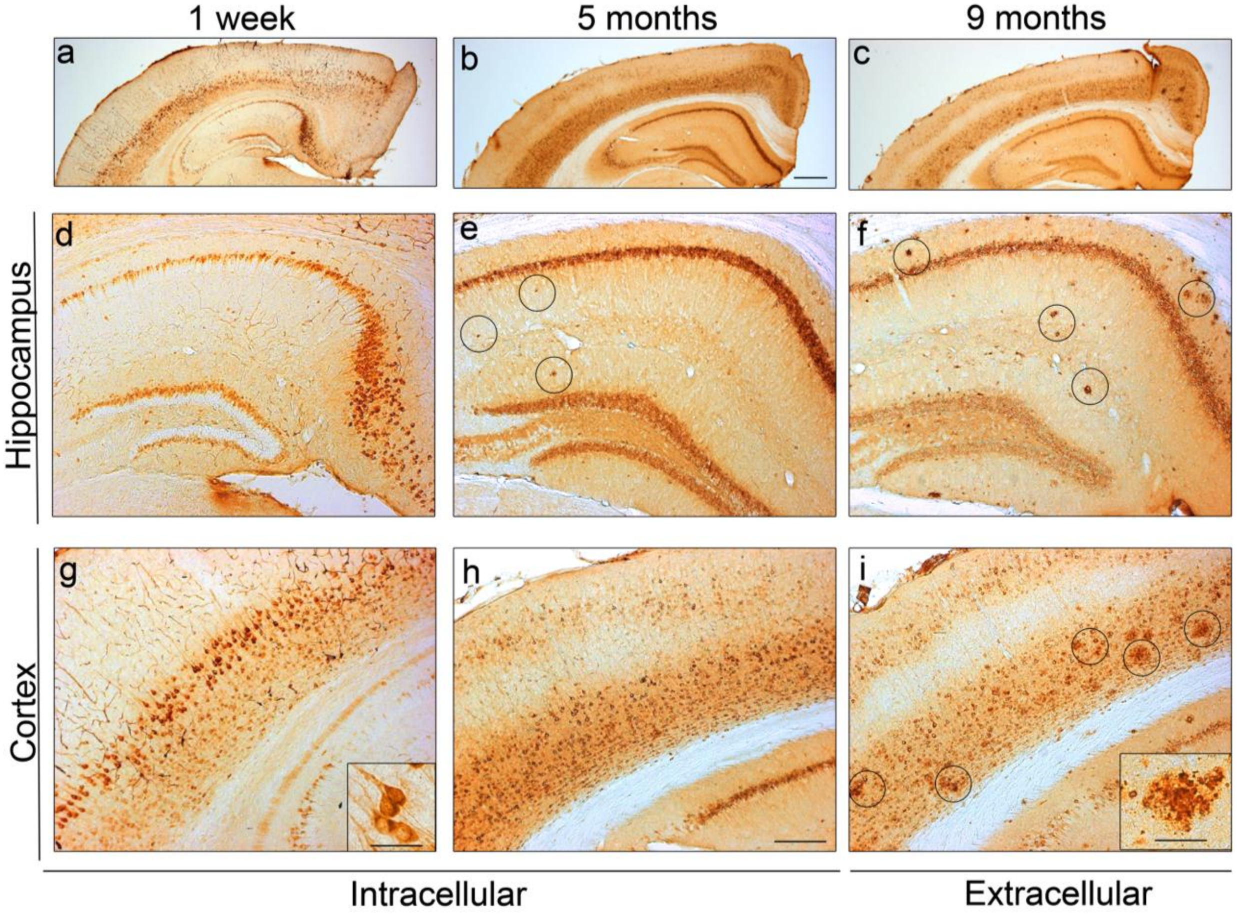

Supplementary Figure S1. Characterization of the amyloid pathology in the McGill-Thy1-APP Tg mouse. (**A**) McSA1 immunoreactivity indicates a pronounced intracellular accumulation of Aβ in the cerebral cortex and hippocampus of 1 week old Tg mice. (**B**) 5 month-old Tg mice show a mixed pathology with the appearance of diffuse amyloid plaques. (**C**) 9 month-old animals display a more advanced pathology showing both intracellular immunoreactivity and extracellular plaque deposition. (**D,E,F**) higher magnification micrographs illustrating Aβ- immunoreactivity in the hippocampus. Note the plaque deposition (black circles) at 5 and 9 months (**E,F**). (**G,H,I**) higher magnification micrographs illustrating Aβ-immunoreactivity in the cortex. Note the neuronal intracellular Aβ (**G**, inset) and a prototypical amyloid plaque (**I**, inset). Scale bar: 500 µm (low magnification), 200 µm (high magnification), 50 µm (inset).

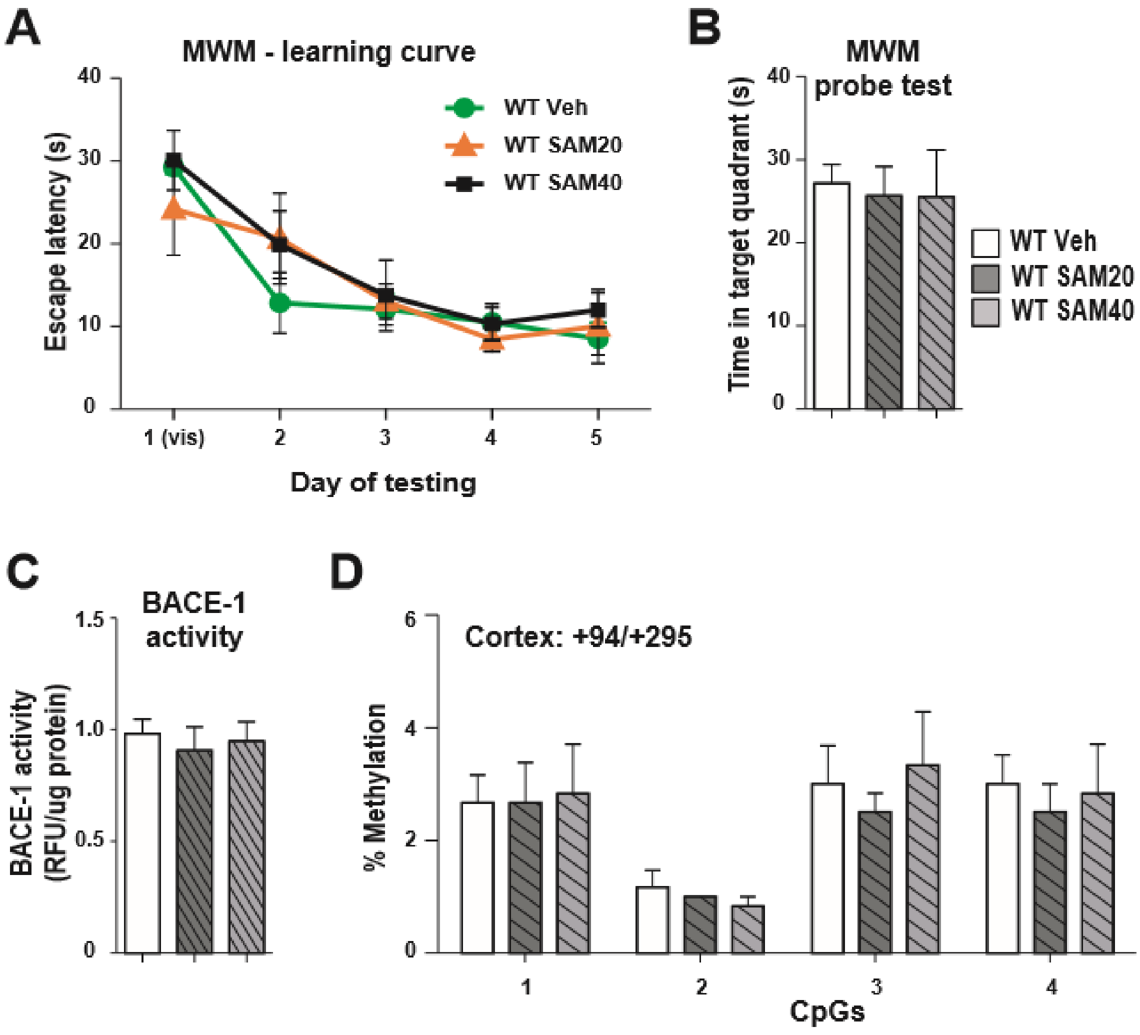

Supplementary Figure S2. Chronic administration of SAM did not impact cognition or BACE-1 in WT animals. (**A**) SAM administration did not impact the learning rate (**A**) or memory recall (**B**) of WT mice in the Morris water maze. (**C**) SAM administration did not affect BACE-1 activity levels as determined by an enzymatic assay. (**D**) Cortical methylation levels of *bace-1* proximal promoter were not affected by SAM treatment. Data are expressed as mean ± SEM and analyzed with One-Way ANOVA, followed by Bonferroni post hoc test.

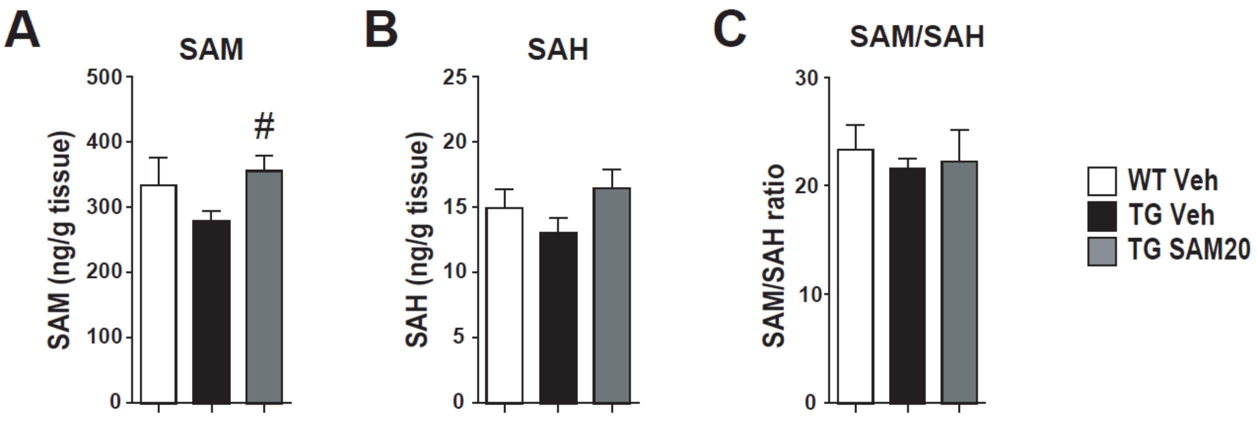

Supplementary Figure S3. Quantification of SAM and SAH levels in Tg animals following chronic SAM administration. (**A**) Chronic administration of low levels of SAM resulted in increased SAM levels in the brain. This was accompanied by a trend towards increased SAH levels (**B**), resulting in an unchanged SAM/SAH ratio (**C**). Data are expressed as mean ± SEM and analyzed with One-Way ANOVA, followed by Bonferroni post hoc test; #: $p<0.05$ vs Tg Veh.
